# Experiences, needs, and perceptions of paternal involvement during the first year after their infants’ birth: A meta-synthesis

**DOI:** 10.1371/journal.pone.0210388

**Published:** 2019-01-07

**Authors:** Shefaly Shorey, Lina Ang

**Affiliations:** Alice Lee Centre for Nursing Studies, Yong Loo Lin School of Medicine, National University of Singapore, National University Health System, Singapore, Singapore; Centre Hospitalier Universitaire Vaudois, FRANCE

## Abstract

**Objectives:**

Fatherhood has evolved in recent decades from ‘a moral teacher’ to ‘an involved father’. However, fatherhood experiences have not been examined in detail. This meta-synthesis aimed to examine fathers’ experiences, needs, and perceptions of their involvement with their infants during the first 12 months of birth.

**Method:**

Six electronic databases were systematically searched: PubMed, CINAHL, Embase, Scopus, PsycINFO, ProQuest (grey literature). The search resulted in 13 studies that met the inclusion criteria. Quality appraisal was conducted using the Critical Appraisal Skills Programme Checklist. All 13 studies met the appraisal criteria and were included in the meta-synthesis. The findings of the 13 studies were synthesized using the steps of Sandelowski and Barroso in conducting meta-synthesis.

**Results:**

The 13 included studies comprised studies conducted in the West, Africa, and Asia. Fatherhood experiences differed according to different sociocultural contexts. Three themes were identified: (1) trajectory of the father-infant relationship, (2) reinforcements and hindrances to involvement, and (3) change from self-oriented to family-oriented behavior. Changes in a father’s relationship with his infant were influenced by relationships with his spouse and family members after the birth of the infant. Reinforcements, hindrances, and needs to fathers’ involvement were identified. Fathering responsibility and parenting satisfaction that developed overtime influenced a father’s behavior, changing from self-oriented to family-oriented.

**Significance:**

This is the first meta-synthesis that examined fathers’ experiences, needs, and perceptions of their involvement with their infants during the first 12 months of birth. Multiple factors were found to influence the socially-defined fathering ideology. Cultural practices of fatherhood and fathers’ own perceptions shaped their actual fathering behaviors. The findings of this study may guide healthcare professionals as frontline personnel to understand fathers’ needs and experiences in order to promote fathers’ involvement in the early days after their infants’ birth.

## Introduction

Fathering behavior is largely shaped and influenced by social changes and cultural contexts. Social expectations of fathering behavior differed in different historical periods, also known as the ‘epochs’ of fathering [1]. In the United States of America, fatherhood over the recent decades has evolved from being ‘a moral teacher’ to ‘a providing father’ and ‘an involved father’ [1–4]. Prior to industrial revolution, the responsibility of fathers was teaching moral values to their children and meeting the educational needs of their children. During the period of industrial revolution and the onset of the division of labor, fathers were accorded the role of being the breadwinner to provide for their families while mothers were the primary caregivers. In examining contemporary fatherhood, fathers are portrayed as ‘involved fathers’ or ‘new men’ who assume the breadwinning role and who are also involved in the family life [2, 5]. ‘Involved fathers’ or ‘new men’ are defined as fathers who continue to assume the breadwinning role due to cultural expectations and, at the same time, play a more involved role in caring for their children [2, 5].

The traditional gendered roles of men being the breadwinner and women being the caregiver were altered during the period of industrialization. More women were engaged in paid work outside their families, which gave rise to rising dual-income households and a change in the cultural image of fatherhood in developed countries. LaRossa highlighted that there exists a mismatch between the culture and conduct of fatherhood in the United States of America [4]. The conduct of contemporary fathers does not match the cultural expectation of an involved father. Fathers were aware of the ideal types of fatherhood they were expected to play; such as being involved fathers or new men. However, they did not behave according to those ideal types, resulting in the mismatch. Guided by the concept of reflexivity (whereby individuals shape their own choices, norms, and behaviors), theorists such as Giddens [6] and Beck [7, 8] noted that as family life is influenced by social change, globalization, work changes, and political changes, fathers are free to decide on the fathering roles they wish to take on. On the other hand, Williams [9] took on a different stance in arguing that men are compelled to make decisions on their fathering roles based on a variety of factors rather than choosing the roles they wish to take on. In summary, fathers were aware of what they should do, but what they actually do is a result of a combination of various circumstances that they have no control of.

Culture has an influence on fathering behavior. Despite the shift towards a more egalitarian ideology due to the increasing number of women entering the labor market, Yeung [10] found that Asian fathers were less involved in providing physical and emotional care to their children compared to mothers as the breadwinning role remained to be fathers’ dominant identity in the family context. Family ideology in Asia is heavily guided by patriarchy due to Asian ideological and religious influences from Confucianism, Hinduism, and Islamism. Patriarchy is more dominant among Shanghai fathers in China and Vietnamese fathers in whom Confucian ideology is heavily ingrained. Also, among Muslim Malay fathers in Malaysia, Malay families have always been found to be patriarchal [10]. In Singapore, mothers were the primary caregivers their children along with extended families and domestic helpers, fathers’ central role in the family remains as a provider and were therefore not socially expected to participate in the physical or emotional care of their young children [10, 11]. In India, mothers were found to be responsible for the early childcare of children while fathers were out at work earning money [12]. Influenced by Confucian teachings, mothers were expected to be responsible for childrearing and domestic work while fathers played less of a direct role during infancy and young childhood in Vietnamese families [13]. In China and Hong Kong, there exists a worrying phenomenon related to fathering, as Hong Kong fathers were found to spent an average of six minutes a day with their children and Chinese fathers spent a maximum of 54 minutes a day with their children [14].

Although men are seen to desire to be more involved in the family life, there exists many challenges and difficulties in fulfilling the ideal type of fathering role. Men in the United Kingdom are becoming more involved in family life and taking more responsibility in child care duties. However, they remarked that they were being ‘squeezed’ between playing the role of an involved father and the breadwinner’s role due to work commitments [15]. Work commitment was one of the highest reported hindrance by fathers to be more involved in family life and in child care in other sociocultural contexts [2, 16–18]. Other factors that simultaneously influenced fathering behavior include fathers’ relationship with their own father [19], fathers’ attitudes on the parenting role [16], infant temperament [20], marital relationship, [21] and partner’s employment status [22].

Maternal-infant bonding and synchrony (dyadic interaction) are beneficial for cognitive and social development, physical health, infant attachment, and trust [23, 24]. Early paternal involvement during infancy was also found to benefit infants’ neurodevelopment [25] and yield positive behavioral outcomes for children aged nine and eleven years old [26]. Therefore, fathers were encouraged to be involved as early as during the perinatal period by healthcare professionals. However, the postpartum period is widely known to be stressful and challenging. During this period of time, feelings of joy at the birth of the child can turn into feelings of frustration as the demands of parenthood kick in [27]. Therefore, it is crucial to understand fathers’ experiences and needs during this period, when paternal involvement is shaped. To the best of our knowledge, one meta-synthesis was conducted by Goodman [28] on the fathering experiences of infants, which included peer-reviewed studies from 1990 to 2001. The meta-synthesis included studies that examined fathers’ experiences and perceptions across the perinatal period, and it revealed the process of becoming an involved father through four stages. However, the broad focus of the perinatal period limits the challenges that were pertinent during the postpartum period. Furthermore, the participants of the synthesis included both fathers and mothers, which may have hindered the experiences and needs of fathers being fully accounted for. Lastly, the meta-synthesis included studies from 1990 to 2001, which is backdated and changes to fatherhood may have been omitted.

This meta-synthesis aims to examine qualitative studies on fathers’ experiences, needs, and perceptions of their involvement with their infants during the first 12 months of birth. Meta-synthesis is valuable and beneficial in providing a novel perspective and fuller interpretation of a phenomenon through the compilation of several qualitative studies [29]. Multiple determinants, including sociocultural context, shape fathering behaviors. It is important to understand fathers’ experiences, perceptions, and needs from their perspectives during early infancy so that necessary interventions may be given to fathers during the first 12 months of birth as it is known to be a stressful and challenging period of time.

## Materials and methods

### Search strategy and screening

The reporting guidelines were followed in accordance to the Preferred Items for Systematic reviews and Meta-Analysis (PRISMA) statement (S1 Table) [30]. Six databases were searched for articles that were published between 1990 and 7^th^ May 2018: PubMed, Cumulative Index to Nursing and Allied Health Literature (CINAHL), Embase, Scopus, Psychological Information Database (PsycINFO), and ProQuest (grey literature). According to the purpose of the study, four main concepts were derived: “fathers”, “postpartum period”, “involvement in infant care”, and “experiences or needs”. Varied combinations of search terms and MeSH terms that were unique to each database were used in the search strategy. These search terms included fathers, paternal, husband, postpartum, postpartal, postnatal, infancy, postpartum period, childbirth, infant care, infant duties, child care, involve, engage, interact, play, bath, shower, breastfeed, coo, feed, change, talk, bond, father-infant, skin-to-skin, kangaroo care, infant diaper, postnatal care, postpartum care, experiences, and needs. An example of the search strategy for one of the electronic databases, PubMed, can be found in S2 Table. The search was limited to be identified by titles and abstracts in all the six databases. Additionally, the backward citation tracking method was used to identify eligible articles that may have been missed out during the electronic search. Two of the reviewers screened the titles and abstracts independently for their relevance to the topic of interest. After screening through the titles and abstracts, the full-text of each eligible article was downloaded and assessed according to the inclusion and exclusion criteria for the final inclusion. All discrepancies in the final inclusion of the included articles were discussed and resolved with the moderation of the third reviewer who did not participate in the initial selection and screening process.

**Fig 1:**
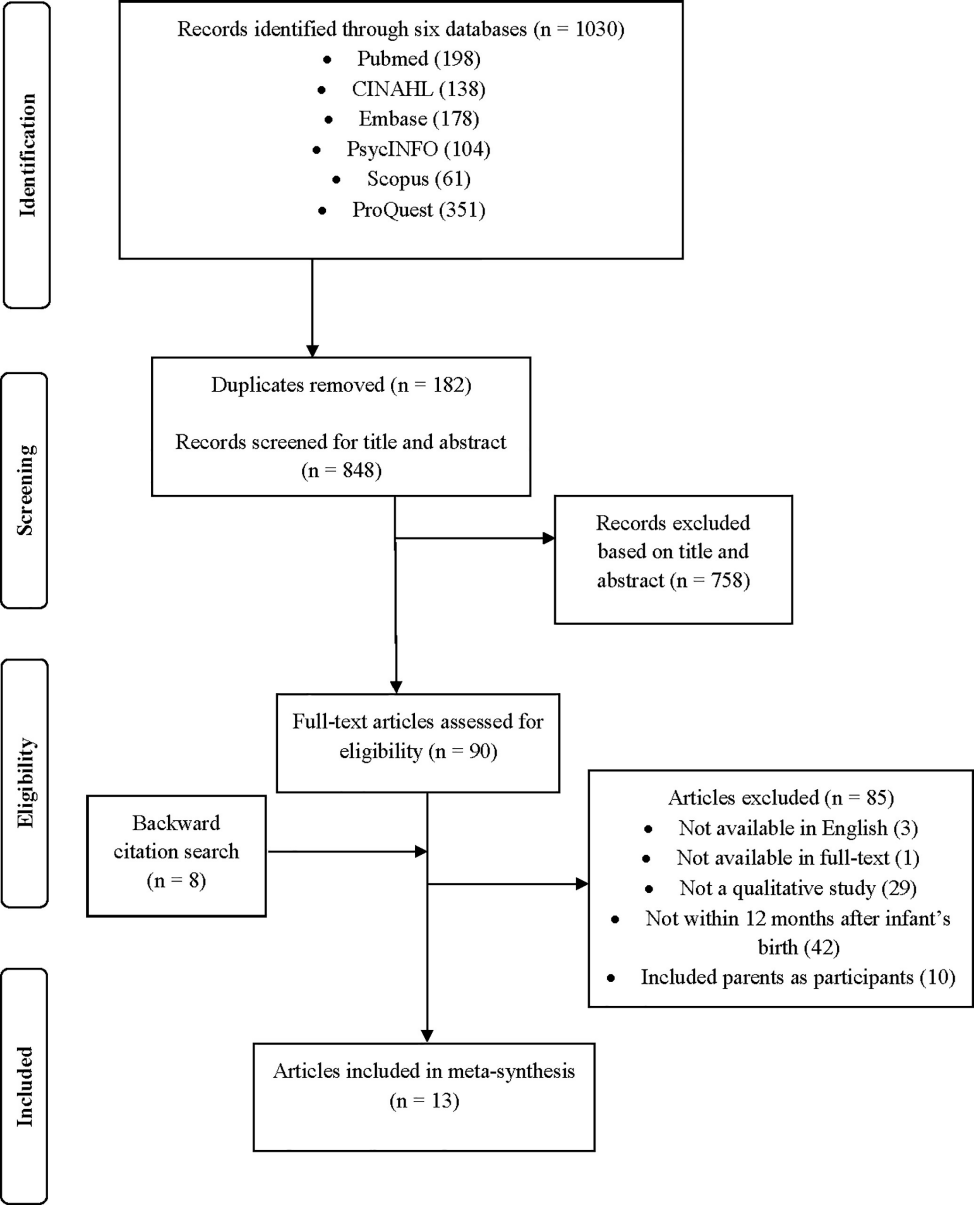
PRISMA flow diagram.

The initial search in the six databases resulted in 1,030 records. After the removal of duplicates and screening by titles and abstracts, 90 full-text articles were downloaded and assessed for eligibility. Backward citation tracking yielded an additional eight articles that were not found through the electronic search. A total of 85 articles were excluded as they did not meet the inclusion criteria. Most of the studies were excluded on the basis that they did not adopt the qualitative methodology and were not studied within the postpartum period of 12 months. The final inclusion in the meta-synthesis was 13 peer-reviewed qualitative studies. Details of the search process and outcomes can be found in Fig 1.

The inclusion criteria were:

1. Population: healthy fathers who were above the age of 18 years old regardless of their ethnicity, social status, and educational level and whose infants were born healthy.
2. Context: from the day of the infant’s birth to 12 months after the birth of the infant.
3. Outcomes: experiences or needs on the involvement of infant care.

Studies that included fathers under the age of 17 years old, who had existing psychiatric or psychological conditions, whose infant were born pre-term or pre-maturely were excluded. Additionally, studies that examined fathers’ involvement in the prenatal period and childbirth only period, studies that examined infants who were older than 12 months, and studies that were not qualitative in nature were also excluded in this review.

### Critical appraisal

The 13 potential studies that met the stringent inclusion criteria were critically appraised for their rigor and quality by the two independent reviewers. The Critical Appraisal Skills Programme (CASP) tool [31] was used to appraise the eligible studies. The tool consists of ten items that assessed if the aims, methodology, design, sampling strategy, data collection, reflexivity of the researchers, ethical consideration, rigor of data analysis, statement of findings, and value of the research were appropriate and clear. Ratings for the ten items were rated as “Yes”, “Can’t Tell”, and “No”, with “Yes” being accorded a score of “1” and “Can’t Tell” and “No” being accorded a score of “0” for each item. The cut-off score for the final inclusion of the studies set by the reviewers was decided to be 8 out of 10 in order to ensure that the included studies were sufficiently rigorous. A score not exceeding ten was given for each of the appraised studies, and the 13 appraised studies were rated an average of 9 by the two independent reviewers. Out of a possible total score of 130 (range: 0 to 130), a score of 122 was given by both reviewers (SS and LA) in the rating of the study quality of the 13 included studies. This means that out of the 130 questions, 122 of them were rated ‘Yes’ by both reviewers. Inter-rater agreement (not weighted) between the two reviewers was approximately 97%. The percentage was calculated based on the number of similar ratings (126) given by both reviewers across the 13 included studies divided by the possible score of 130. All 13 eligible studies passed the critical appraisal and were included in the meta-synthesis. The included studies have clear research aims, valuable findings, appropriate methodology, design, sampling, and data collection method. Ethical issues were addressed, and the data analysis was rigorous. However, information on the reflexivity of the researchers was insufficient or not mentioned in most of the studies. The results of the critical appraisal can be found in S3 Table.

### Data extraction

The included studies were read through several times by the reviewers. In view of the purpose of this meta-synthesis to understand fathers’ experiences, perceptions, and needs from their perspectives during early infancy, the extracted data included fathers’ negative and positive lived experiences, feelings, needs, and challenges faced when caring for their infants during the first year after each infant’s birth. The following data were extracted: details of the study, study aims, methodology of the study, details of data collection and analysis, sample characteristics and significant findings of the study in relation to the study aims. Data extraction from the 13 included studies was conducted independently by the two reviewers using a standard form that contained the following categories: (1) study (author, year, country), (2) aim(s) of study, (3) methodology, data collection, and data analysis, (4) data collection timepoint(s), (5) sample characteristics, and (6) major themes and subthemes. The third reviewer performed data extraction on three of the included studies (23%) for validity. A summary of the included studies can be found in Table 1.

**Table 1:** Summary of the included studies.

Author/ Year/ Country	Aim(s) of the Study	Methodology/ Data Collection/ Data Analysis	Data Collection Timepoint(s)	Sample Characteristics	Main Findings
**Europe**					
Fagerskiold (2007) Sweden	To explore first-time fathers’ experiences during the early infancy of their children.	Grounded theory; semi-structured individual interviews; constant comparative method	When their infants were approximately 5 to 9 months old (not explicitly stated)	20 first-time fathers, 20–48 years old, worked full-time, and partners had parental leave, with infants between 5–9 months old.	First-time fathers experienced a change in life in the aspects of 1) becoming a father, 2) alternating between work and home, 3) changing relationship towards partner, and 4) developing a relationship with their infant. Becoming a father was much more fantastic than fathers could imagine; however, alternating between work and home made them feel that the mother was still the main parent. Their relationship with their spouse became closer. However, tiredness could cause increased irritability towards problems due to the lack of sleep. Developing a relationship with their infant implied learning to know more of their infant’s signals.
Olsson et al. (2010) Sweden	To describe fathers’ experiences about their sexual lives 3 to 6 months after the birth of their children.	Qualitative study (descriptive); 2 focus group discussions and 5 semi-structured individual interviews; content analysis	Three to six months after the births of their babies.	10 fathers (8 first-time and 2 subsequent), 26–51 years old, middle-to-upper class, and partners had normal pregnancies.	The male sexuality is affected during the transition into fatherhood as it brings sexual life to a crossroads. To get their sexual lives working, fathers need to be involved in infant care and household chores and get in tune with their spouses with regard to sexual desire. Healthcare professionals need to reassure and prepare men for this new situation prior to childbirth.
Premberg et al. (2008) Sweden	To explore the experiences of the first year as a father	Phenomenology; semi-structured individual interviews; Giorgi’s phenomenological method	12 to 14 months after the delivery of their first child.	10 first-time fathers, 25–32 years old, and all of them had participated in childbirth education.	Fathers placed the infant in the center of their lives without giving up one’s own person. The addition of the infant brought about happiness and warmth in the family, and fathers experienced a deeper relationship with their spouses. The contact between father and infant was facilitated by engagement and time spent alone with the child. To master fatherhood, the maintenance of integrity and possibility to develop an independent relationship with the infant is important. Healthcare professionals must be aware of fathers’ needs.
Feenstra et al. (2018) Denmark	To explore how new fathers experience early discharge after birth and the readmissions of their newborns in relation to their role and involvement as a father.	Qualitative study (hermeneutics phenomenology); semi-structured individual interviews via telephone; systematic text condensation	Two to five weeks after the newborn’s birthday.	Six fathers (3 first-time fathers), 24–45 years old, and their partners had uncomplicated pregnancies and birth, with healthy newborns.	First-time fathers reported being comfortable with early hospital discharge as the hospital environment was stressful to them; however, they experienced insecurity at home due to the lack of knowledge and infant care skills. Fathers felt opposed that they were classified by healthcare professionals as the practical guy who had to assist their spouse when they felt they were equally important to the mothers. Fathers saw themselves in the shadow of their spouses and had greater considerations for the feelings of their spouses over their own. The role as the practical guy made fathers insecure in their paternal role.
**North and South America**					
Anderson (1996a) Canada	To explore fathers’ experiences of developing relationships with their infants during the first two months of their infants’ lives.	Grounded theory; semi-structured individual interviews; constant comparative method	Approximately two months postpartum (not explicitly stated).	14 first-time fathers, 28–44 years old, Caucasian, middle class, with healthy infants.	Fathers’ commitment to the relationship with their infant took hold at different times. Perceived rewards influenced fathers’ feelings of connectedness towards their infants. The father-infant relationship continues to develop when infants could smile and vocalize. Some fathers experienced ‘hesitant connection’ whereby feelings of love for their infants did not appear at first. Nurses need to reassure fathers that father-infant relationships may evolve more slowly than mother-infant relationships.
Anderson (1996b) Canada	To describe the factors that influence the initial development of the father-infant relationship during the first two months of the infant’s life.	Grounded theory; semi-structured individual interviews; constant comparative method	Approximately two months postpartum (not explicitly stated).	14 first-time fathers, 28–44 years old, Caucasian, middle class, with healthy infants.	Factors that influenced the father-infant relationship were 1) fathers’ adjustment in their work and social time, 2) relationship with their spouse, 3) relationship with their own father, and 4) emotional and informational support from spouse. Mothers were found to have a powerful influence on the father-infant relationship.
de Montigny and Lacharité (2004) Canada	To describe the perceptions of first-time fathers regarding critical moments of the immediate postpartum period.	Qualitative study (critical incident technique); semi-structured interviews; critical incident technique	Within the first 12 days after infant’s birth.	13 first-time fathers, French-speaking, mean age of 30.5 years old, with healthy infants.	Three groups of fathers were found depending on their presence in the maternity ward: 1) less involved fathers, 2) moderately involved fathers, and 3) highly involved fathers. All three groups of fathers were proactive and conscious to interact with their infants and nurses during the first few days of postpartum. Highly involved fathers did not feel supported by the hospital environment (nurses and policies) in being involved with their infants.
Gamble and Morse (1993) Canada	To examine husbands’ experiences of having their wives breastfeed.	Grounded theory; semi-structured individual interviews via phone; simultaneous process of data collection and analysis guided by the grounded theory	5 participants were interviewed once, 7 were interviewed twice, and 2 were interviewed thrice within the period of 3–6 months postpartum.	14 middle-class, urban, Canadian fathers (8 first-time fathers, 5 have 2 children, and 1 had 3 children), 22–35 years old, with infants breastfed 3 to 6 months.	Fathers found a disparity in the relationships that their infants had with each parent due to breastfeeding. ‘Labeled postponing’ is a process that enabled fathers to accept the perceived difference in the relationships. The phases of this process include: 1) becoming aware of the disparity, 2) simultaneously developing accepting strategies and acknowledging reinforcing factors, and 3) developing compensating behaviors to increase fathers’ interactions with their infants and promote closer relationships. Fathers found ways to catch-up in their relationships with their infants when weaning occurred.
Ayala et al. (2016) Chile	To describe fathers’ experiences and perceptions of being the primary caregiver to their newborn infants during the first 90 minutes after caesarean section.	Qualitative study; a questionnaire with four open ended questions related to the aims of study; systematic text condensation	90 minutes after the infant’s birth through an uncomplicated caesarean section.	95 fathers, average age of 32 years old, Chilean origin, with an average of two previous children, with healthy focal infants.	After 90 minutes of caring for their newborn infants, fathers found that the process brought about feeling of love for the infants and they felt closer bonds with them. Fathers also reported that the process was beneficial to the infants, their spouses, themselves, and the future of the family. They recommended this caring model in which fathers were the primary caregiver of their infants during the first 90 minutes, not just for caesarean section birth but also for normal birth.
**Australia**					
John et al. (2005) Australia	To explore new/subsequent Australian fathers’ perspectives on the experiences, processes, and life changes in the early weeks of fatherhood.	Grounded theory; in-depth interviews; constant comparative analysis	6 to 12 weeks after the birth of each infant.	18 fathers (first time and subsequent).	Fathers found new or expanding fatherhood to be both rewarding and challenging. Work commitments limited fathers’ abilities to participate with their families and newborns. Fathers had to balance the demands of work and demands at home. They have to deal with varied stressors, manage their time, develop routines and reprioritize their tasks at hand. Fathers developed a sense of self as fathers over time, building their confidence and satisfaction in the fathering role.
**Africa**					
Mbekenga et al. (2011) Tanzania	To explore postpartum experiences of first-time fathers in a multicultural, low-income suburban Tanzanian setting.	Qualitative study (interview); semi-structured individual interviews; content analysis	4 to 10 weeks postpartum.	10 first-time fathers, 24–34 years old, who perceived themselves and their infants to be healthy and are living with the mothers of the infants.	First-time fathers reported enjoying fatherhood. They were active in mother and infant care and household chores but were limited by breadwinning responsibilities. Traditions prescribed abstinence to sex while the woman is breastfeeding, which made the timing of resuming sexual activity after childbirth problematic. Fathers were found to be excluded by healthcare professionals and information given by them were often unclear.
**Asia**					
Shorey et al. (2017) Singapore	To explore first-time fathers’ postnatal experiences and support needs in the early postpartum period.	Qualitative study (descriptive); semi-structured individual interviews; thematic analysis	Approximately 5 to 14 days after their partner’s date of delivery.	15 first-time fathers, mean age of 31 years old, married with a mean period of 2 years.	Fathers of different races have varied unmet needs in the early postpartum period such as sleep-deprivation and being unaware of the available educational support. Fathers were confused and lacked confidence in the paternal role. Fathers reported feeling lost and desired to be involved in postnatal educational programs. They also showed preferences towards electronic media such as mobile-health applications for receiving information online.
Shorey et al. (2018) Singapore	To understand fathers’ expectations, needs and experiences in infant care during the early postpartum period.	Qualitative study (descriptive); semi-structured individual interviews; thematic analysis	One week after the birth of the focal infant.	50 fathers (34 first-time fathers and 16 experienced fathers), 22–50 years old, multi-ethnicity.	The postpartum period was found to be stressful for both first-time and experienced fathers. Fathers desired to be involved but were hindered by their spouses who had little confidence in their infant care skills, work commitments, and lack of infant care skills. Experienced fathers faced the challenge of assimilating older children with newborn infants.

### Data synthesis

This meta-synthesis followed the two stages of synthesizing qualitative research by Sandelowski and Barrosso [32]. The first stage of data synthesis was performed by meta-summarizing the findings of the included studies into statements. Fathers’ experiences, perceptions, feelings, needs, and challenges faced during the first year after their infants’ birth were categorized and thematized into main themes using the procedure of thematic analysis illustrated by Braun and Clarke [33] by the two independent reviewers. Consensus on the main themes was reached with the help of the third reviewer before conducting the final meta-synthesis. Effect size was computed for each theme by dividing the number of studies containing the theme with the total number of studies included in this meta-synthesis [34]. The second stage of the data synthesis went beyond merely summarizing and describing the data to generate new or expanded theories or concepts [35]. A novel perspective and understanding of fathers’ experiences and needs could then be derived through the interpretation of the pooled findings. The constant targeted comparison [32] between each study was used to synthesize the meta-summary produced in the first stage of data synthesis. This constant comparison was able to identify overlapping similar attributes of the phenomenon and reveal new understanding of fathers’ experiences and needs in the first year after the birth of their infant. Triangulation was maintained through a constant targeted comparison of the meta-summary with individual studies.

## Results

### Characteristics of the included studies

Of the 13 included studies, ten [36–45] of the studies were conducted in the West, two [46, 47] of the studies were based in Asia, and one study [48] was conducted in Africa. Of the ten studies conducted in the West, four studies [40, 41, 43, 44] were conducted in Europe (Denmark and Sweden), five studies [36–39, 42] were conducted in North and South America (Canada and Chile), one study [45] was from Australia, one study [48] was conducted in Africa (Tanzania), and the two Asian studies were conducted in Singapore [46, 47]. Five studies [36, 37, 40, 42, 45] adopted the grounded theory methodology, seven studies used the qualitative study design which consisted of the descriptive [43, 46, 47], critical incident technique [39], open-ended questionnaire [38], phenomenology [41, 44], and interview [48] approaches. Semi-structured individual interviews were mainly used for data collection, and a variety of data analysis method, comprising constant comparative method, systematic text condensation, critical incident technique, content analysis, Giorgi’s phenomenological method, and thematic analysis, were used. The sample size ranged from six to 95 first-time and subsequent fathers, totaling up to 289 fathers.

The included studies showed that the fathering role is less articulated compared to the mothering role and that fathering behavior is shaped and influenced by multiple determinants. A combination of factors, such as socially-defined fathering ideology, cultural practices of fatherhood, and fathers’ own perceptions, influenced fathering roles and shaped their actual fathering behaviors. ‘Fatherhood: a roller coaster ride’ emerged as an overarching theme from the meta-synthesis in understanding the experiences and perceptions of early fatherhood from fathers’ perspectives. The overarching theme was supported by three subthemes that emerged from the data analysis: (1) trajectory of the father-infant relationship, (2) reinforcements and hindrances to involvement, and (3) change from self-oriented to family-oriented behavior. The themes are discussed and supported by the participants’ quotes from the studies that they were extracted from. The presence of key aspects contributing to synthesized themes are presented in the summary of findings Table 2.

**Table 2:** Summary of the findings.

Themes	Definition of theme	References
1) Trajectory of the father-infant relationship	Changes of the father-infant relationship during the first year after the infant’s birth and how the relationship changes overtime	Anderson, 1996a [37]; Anderson, 1996b [36]; Ayala et al., 2016 [38]; de Montigny & Lacharité, 2004 [39]; Fägerskiöld, 2008 [40]; John et al., 2005 [45]; Premberg et al., 2008 [44]; Shorey et al., 2017 [46]; Shorey et al., 2018 [47]
2) Reinforcements and hindrances to involvement	The factors that hinders and reinforces paternal involvement in the care of the infant	Anderson, 1996a [37]; Anderson, 1996b [36]; Ayala et al., 2016 [38]; de Montigny & Lacharité, 2004 [39]; Fägerskiöld, 2008 [40]; Feenstra et al., 2018 [41]; Gamble & Morse, 1993 [42]; John et al., 2005 [45]; Mbekenga et al., 2011 [48]; Olsson et al., 2010 [43]; Premberg et al., 2008 [44]; Shorey et al., 2017 [46]; Shorey et al., 2018 [47]
3) Change from self-oriented to family-oriented behavior	The process of change in a father’s behavior from self-oriented to family oriented	Anderson, 1996a [37]; Anderson, 1996b [36]; Ayala et al., 2016 [38]; de Montigny & Lacharité, 2004 [39]; Fägerskiöld, 2008 [40]; Feenstra et al., 2018 [41]; Gamble & Morse, 1993 [42]; John et al., 2005 [45]; Mbekenga et al., 2011 [48]; Olsson et al., 2010 [43]; Premberg et al., 2008 [44]; Shorey et al., 2017 [46]; Shorey et al., 2018 [47]

### Trajectory of the father-infant relationship

Changes in relationship with the infant were seen during the first year of birth. The father-infant relationship grew closer and stronger over time when father-infant bonding strengthened through the father getting to know the infant and spending time and having physical contact with the infant [36, 38, 45]. Father-infant relationships were found to establish two months after the infants’ birth when the infants were able to interact with their fathers at this developmental stage [36, 45]. Fathers got to know their infants through comparing their and their spouses’ physical attributes with their infants’ physical attributes: “*His eyelashes are growing in really strongly*, *and both my wife and myself have very long eyelashes*, *so that’s showing up” (Canadian father)* [36] (p. 89). Additionally, physical contact with the infant helped to develop physical bonding, as one father reported: *“I didn’t love her right away when she first came out*. *I think you have to develop that*, *you know*, *she’s part of you*, *and you feel a bond with that*, *but the more you get to know her*, *hold her*, *and handle her”(Canadian father)* [36] (p. 87). However, there were some fathers who reported that they fell in “love at first sight” when they saw their infants for the first time and felt a sense of joy and happiness [38].

The trajectory of fathers’ relationships with their infants was strengthened or deteriorated during the early postpartum period because of the changes in their relationships with their wives and other family members. Fathers communicated with their spouses about infant care: *“I talked with Roxanne*. *I was learning with her*, *so I asked her my questions [about infant care]” (Canadian father)* [39] (p.333) and *“We talk a lot*, *as we always have*. *We did it before we had a child*, *so I don’t think we have changed our relation*. *We talk a lot and discuss problems at an early stage” (Swedish father)* [44] (p.60). Fathers reported that the demands of infant care and the busyness of life affected their personal time with their spouses: *“Life has become somewhat divided*. *We don’t have as much time for each other… Our little girl is in the center of things until nine at night…” (Swedish father)* [40] (p.67). One father reported his resentment of the infant’s intrusion on the close marital bond with his spouse: *“I’m used to being the number one person and I’ve walked in and my wife*, *literally… and now*, *it’s like you’re kind of second…” (Canadian father)* [36] (p.88). One father reported the internal stress he faced when his spouse forced him to be more involved: *“I’m forced to wear different hats* … *handyman*, *breadwinner*, *and gardener… and now*, *it’s increased to housekeeper*, *emotional support person*, *social worker sometimes… and I’m no good at that… but I’m expected to be good at it…” (Canadian father)* [37] (p.316-317).

Fathers’ relationships with their family members were found to vary across different cultures. In Western culture, a baby was a source of joy to grandparents and extended family and fathers themselves were happy with such relationships [44, 45]. Singaporean Chinese fathers were satisfied with the instrumental help provided by family members, but stress and tension arose when they felt pressured to obey the “cultural folklores and confinement practices” instituted by their elders during the postpartum period [46, 47]. Fathers reported the conflicting views: *“For traditional Chinese confinement*, *it’s like you cannot get off the bed and you cannot open the window… So*, *you cannot wash your hair at first*. *In Singapore*, *the weather (is hot) after 1 or 2 days*, *it (hair) will smell*. *It’s not good for me and not good for my wife and my child” (Singaporean father)* [46] (p.2992) and *“Sometimes*, *the philosophy (is) a bit different… My mother-in-law and I had tension regarding breastfeeding*. *She didn’t breastfeed in the past and pushed the same for my baby*. *So*, *I mean*, *I was quite insistent [on breastfeeding by my wife]*, *to the extent of*, *like*, *quarrelling with her (my mother-in-law)” (Singaporean father)* [47] (p.32).

### Reinforcements and hindrances to involvement

A variety of factors were found to encourage fathers’ involvement in caring for their infants. Most fathers reported that their relationships with their own fathers (whether close or distant) affected their desires to be involved fathers both in Western and Asian contexts [37, 41, 45, 47, 48]. Fathers reported: *“My father was fetching water… He carried water on his head*. *He fetched firewood… I was really asking God to live that kind of life*.*” (Tanzanian father)* [48] (p.176) and *“My own father did not participate very much and was very occupied with working… so I try to participate in as much as possible from when they are newborns*, *because I want a good relationship with them” (Danes father)* [41] (p.66). Fathers were also motivated to care for their children through the rewarding experiences of playing with [36, 47], taking care of [39], and holding their infants [38]. Fathers expressed feelings of joy and closeness during these times: *“She can focus now*, *and she can see my face and grin at me and that sort of thing*, *and so I feel she recognizes me*, *and I’m pulled in*, *and I want to play longer and do things longer” (Canadian father)* [36] (p.89), *“I feel closer to my baby*, *because of these experiences (bathing*, *feeding*, *and diaper-changing)” (Canadian father)* [39] (p.332).

Support from their spouses to be involved with their infants spurred Western fathers’ involvement according to fathers: *“She’s (spouse) very good at making people feel confident in themselves… so I don’t feel like I am messing up… I feel confident around the baby” (Canadian father)* [37] (p.318) and *“My partner enjoys being at home with the baby*, *but she wants equality… that I shall take parental leave” (Swedish father)* [40] (p.67). However, fathers in Singapore reported that they did not receive any form of support or encouragement from their spouses: *“Most of the time*, *things are done by my wife and mother-in-law… so*, *for me*, *yes*, *maybe I would like to learn*, *but I don’t think I need to…” (Singaporean father)* [46] (p.2993) and *“I didn’t find anyone who gave me feedback on how I was doing with my role as a father… I was confused…” (Singaporean father)* [47] (p.32).

Other deterring factors of fathers’ involvement were work commitments [37, 40, 44, 45, 47, 48], sleep-deprivation [39, 46, 47], and lack of infant care skills for first-time fathers [38, 46, 47]. Fathers felt that the need to undertake the provider’s role for the family hindered them from spending time with their infants. One father shared: *“(The newborn) makes me want to stay home more… and I have to go out (to work)” (Canadian father)* [37] (p.184). The feeling of alienation from the close relationship of the mother-infant dyad was present according to one father: *“At first*, *I felt like it was her baby… She could be at home with him whereas I worked… I felt very outside…” (Swedish father)* [40] (p.67). Sleep deprivation and the lack of infant care skills resulted in fathers being scared of the early postpartum period and taking care of their infants, as reported by fathers: *“I am scared and skeptical if I want a second child*, *but I think the… the experience of the last two weeks*, *uh*, *I am quite happy to just stop at one” (Singaporean father)* [47] (p.33) and *“When I see the baby is so fragile… I think a cot or bed is better than holding the baby… I can maybe harm the baby” (Chilean father)* [38] (p.77).

Fathers of breastfed infants felt that they had ‘limited access’ to their infants as they were ‘tied to the mother’s apron strings’ [42, 44]. During the breastfeeding process, fathers felt ‘insignificant’ and ‘distant’ from their infants due to the lack of father-infant opportunities [40, 44, 48]. One father felt limited in what he could do during the first few months: *“You’re limited in what you can provide at that point… You can hold her and cuddle her*, *and you can change her diapers*, *and it stops there those first few months when she’s totally dependent and completely breastfed” (Canadian father)* [42] (p.359). At the cessation of breastfeeding, fathers felt the need to develop the father-infant relationship through being more involved, this process is known as ‘catching-up’ [42, 45, 48]. Fathers expressed their desires to be on par with their spouses in their relationships with their infants: *“Well*, *I’m still catching up*. *I think she’s still got the edge on me on putting him to sleep and feeding him…” (Canadian father)* [42] (p.361) and *“I joked about this for the first little while*, *his mother came first*, *then most of the walls in the house and the fireplace*, *and I was like a distant fifth or sixth behind anything*. *But now*, *I’m sort of moving up gradually*. *I think I’m ahead of the fireplace now [laughs]” (Canadian father)* [36] (p.88).

Fathers desired to be involved during the early postpartum period, but they were excluded by healthcare professionals. Fathers were seen as ‘assistants’, ‘helpers’, and even ‘bystanders’ by healthcare professionals during their stays in the hospital [39, 41, 46]. Fathers felt ‘distanced’ when they were not allowed in the labor and postpartum wards, and [48] they felt ‘left out’ when nurses focused only on their spouses and infants [41, 46]. One father reported: *“I wanted others to be able to recognize my involvement by simply talking to me*, *by including me in conversations… They (nurses) never asked me how I felt as a dad” (Canadian father)* [39] (p.443). Most of the fathers iterated the need for more information on infant care, breastfeeding, common infant illnesses, signs of what are normal and abnormal, and on sexual life after childbirth [39, 41, 43, 46–48]. One first-time father reported the need for more information to be given during the hospital stay so that readmission could be prevented: *“If we have had some more information during the time at the maternity ward… if the time was better utilized with guidance” (Danes father)* [41] (p.65). Fathers were also confused by the conflicting infant care instructions received from elders in their families and from different nurses [39, 46, 48]. Fathers shared their experiences of the information received: *“My baby has jaundice*, *so the doctor was telling us not to go down and allow some sunshine on the baby because it dehydrates the baby… but*, *for my parents*, *they say the traditional way is to bring downstairs (in the sun)…” (Singaporean father)* [46] (p.2991-2992) and *“Information given by nurses is often different from one nurse to the next*, *sometimes even contradictory… One would tell us to wash the infant like this*, *we would do so*, *and then another one would say no*, *not like this… We didn’t know which one to listen to” (Canadian father)* [39] (p.333).

### Change from self-oriented to family-oriented behavior

Changes in fathers’ behaviors from being self-oriented to family-oriented were found in most fathers in this meta-synthesis. Western and Singaporean first-time fathers reported that they wanted to become responsible fathers [36, 38, 45–48]. One first-time father reported: *“The first one [infant] changes your whole lifestyle*, *because although you are married*, *you have nothing to worry about*, *no responsibility… then all of a sudden up comes the handbrake… You now have responsibility*.*” (Australian father)* [45] (p.183). Fathers expressed the need to protect and love their infants due to their vulnerability and helplessness [36, 37], and it was the feeling of protection that drew fathers closer to their infants. One father felt the need to protect his infant: *“You feel closeness*. *You want to protect her*. *You realize how helpless she really is… A baby is absolutely helpless*, *and you feel the need to protect her” (Canadian father)* [37] (p.315). The involvement of fathers after the birth of their infants also contributed to fathers’ feelings of becoming family oriented. Such involvement included skin-to-skin contact with their infants immediately after childbirth [38], taking care of their spouses and infants in the hospital 48 to 36 hours post-birth [39], and taking on the ideal fathering role of being a provider and caregiver simultaneously [45]. On skin-to-skin contact, fathers expressed that it was ‘very good’ and ‘something special’. One father expressed his joy: *“I felt excellent… It was the most beautiful experience of my life to take care of my little girl*.*” (Chilean father)* [38] (p.77). One father reported the excitement derived from his fathering role despite it being a tough period of time: *“It’s fun*, *completely amazing*. *It’s so exciting*, *everything… Tough too*, *but more exciting than tough” (Swedish father)* [40] (p.66).

Fathers altered their lifestyles in order to ‘make room’ for their infants and spouses through ‘reprioritizing’ personal and social time and hobbies [36, 37, 44, 45] and ignoring their own feelings [41, 42] and sexual needs [43, 48]. Leisure time and hobbies were postponed in order to spend time with their children [44, 45], and how fathers used to live in the past were changed in order to be physically and emotionally present for their infants [36, 37]. A father remarked: *“Once the child is born*, *you have to change the way you live or the way you used to live and accept another person into your way of life…” (Canadian father)* [36] (p.90). Fathers were found to intentionally neglect their feelings so that they could focus on their spouses and infants, as one first-time father reported: *“She was way too nervous*, *and she was crying… At some point*, *they both were crying (i*.*e*. *mother and newborn)… That*, *I think*, *was hard… but I tried to keep calm and stay in control*, *so that I did not say anything stupid” (Danes father)* [41] (p.66). Fathers were seen to suppress their sexual needs for the well-being of their infants and spouses. One father reported that sex could be postponed and he was willing to wait: *“When you start having a functioning sexual life again and it starts being frequent… you can’t expect that everything should be just like before* … *After all*, *it [childbirth] implies quite a big bodily change” (Swedish father)* [43] (p.721). Disengagement in sex was also found to be influenced by cultural beliefs in order to produce a healthy child, as one father noted: *“The child has no strength*. *He is weak*, *as if he is sick… Mucus flows from the mouth all the time*. *He is floppy and weak… Those sperms will get into the breast*, *and the milk is contaminated*!*” (Tanzania father)* [48] (p.177). The identity of a responsible father was established over time, and one father commented: *“Well*, *you grow up a little*. *Now*, *playing for fun is done*. *I’m a daddy” (Swedish father)* at approximately one year after his infant’s birth [44] (p.59).

## Discussion

This synthesis supported Williams’ argument that fathers are compelled to make a decision on their fathering roles based on a variety of factors rather than choosing which fathering role to take on [9]. Fatherhood is influenced and shaped by sociocultural contexts; therefore, fathering behaviors were not entirely the same in the West, in Asia, and in Africa. Reinforcements and hindrances to fathers’ involvement were not completely the same in these regions. However, one common hindrance to fathers’ involvement across the three regions was fathers’ work commitments. Fathering behaviors and the levels of fathers’ involvement are influenced by fathers’ interactions with infants, marital relationship quality, spousal and familial support, work commitments, breastfeeding, the role of healthcare professionals, fathers’ relationships with their own fathers, fathers’ levels of infant care skills, and the interplay of feelings and attitudes. Father-infant relationships as well as fathers’ sense of responsibility were strengthened and crystalized overtime through interactions and skin-to-skin contact with their infants [36, 38, 39, 45]. Fathers’ desires to become involved fathers (one that assumes the breadwinning role and, at the same time, care for their infant) increased overtime through the rewards of experiencing joy and closeness when fathers interact with their infants [28, 36]. Therefore, paternal skin-to-skin contact should be encouraged especially after an infant’s birth as it helps to strengthen father-infant bonding and results in beneficial infant and paternal outcomes [38, 49]. Marital relationship quality and spousal support influenced fathering behaviors. Fathers who reported good martial relationships with their spouses were found to engage in co-parenting behaviors through communicating with their spouses to cope with the demands of early parenthood [39, 44, 45]. Spousal support spurred fathers’ involvement in increasing fathers’ confidence in performing infant care duties [37]. Good marital relations and spousal support were found to positively influence fathers’ involvement as the appropriate fathering role behavior was crystalized [50]. On the other hand, marital conflict was found to reduce fathers’ engagement with their infants and co-parenting styles were less democratic [51]. A quasi-experimental study revealed that fathers who attended father-focused discussion classes on stress, coping strategies, social support, and spousal relations significantly increased their use of reasoning during spousal conflicts and housework activities compared to fathers who attended traditional childbirth classes [52]. Therefore, co-parenting behaviors and marital conflict resolution should be educated to new parents during the antenatal period. The intrusion of their infants and being forced to be involved by their spouses were reported by fathers as reasons of their deteriorating marital relationships. The lack of couple time [40, 45] and the sudden added responsibility that fathers were forced to take up created stress and discontentment in their fathering roles [37]. Spousal support to be involved in caring for their infants was found to be unique to the Western context as Singapore studies revealed the presence of maternal gatekeeping due to the perceived lack of infant care skills by mothers [47]. Fathers in Singapore were not expected to provide physical and emotional care for their infants by their spouses and family members, and their involvement was thus hindered [46, 47]. Fathers felt that their participation was not needed and were confused about the fathering role.

Family members were highly involved in caring for their infants in Singapore, unlike in the West where family life was seen to be independent of extended families and relatives [45]. Another source of fathers’ stress arose from relationships with family members; in particular, in the Singapore context where the help of family members was engaged during the confinement period [10, 46, 47]. Familial relation was less joyous between Chinese fathers and their elders (parents or parents-in-law) due to the conflicting views of cultural confinement rituals. Tension due to differing philosophy on how the infant should be taken care of was found in familial relation of Singapore Chinese families [46, 47]. Aside from cultural practices and beliefs, unique to the Singapore context is the socially-defined father’s role of being a provider and breadwinner of the family [10, 46, 47]. Fathers were not expected to be involved in the care of their infants by their spouses and members of the family despite their desires to be involved [46, 47]. These are sociocultural factors that hindered fathers’ involvement, which are unique in the Singapore context. Other hindrances of fathers’ involvement included work commitments, sleep-deprivation, and lack of infant care skills. Fathers found it challenging to juggle with the provider’s role and trying to be nurturing fathers due to work commitments that took away their time with their infants. Increasing the duration of paternity leave and the possibility of flexible work hours during the early postpartum period could be helpful to new parents during this transition period [47]. Fathers’ lack of infant care skills resulted in maternal gatekeeping and the fear of taking care of their infants, which reduced their involvement [38, 47]. As such, fathers should be equipped with infant care skills and expectant knowledge of the early postpartum period during antenatal classes. Breastfeeding is a unique factor that is both a reinforcement and hindrance to fathers’ involvement. Fathers’ involvement was not absent but delayed as fathers saw the need to ‘catch-up’ in their father-infant relationships to that of mother-infant relationships after their infants were weaned [42]. The process of breastfeeding shaped fathers’ attitudes and desires to build their father-infant relationships at a later stage. Ways of involving fathers even when the child is being fully breastfed should be encouraged.

Healthcare professionals are recommended to act as frontline personnel to increase fathers’ involvement by intentionally engaging and encouraging fathers to be involved through skin-to-skin contact and also providing fathers with standardized information on infant care. Healthcare professionals were found to indirectly exclude fathers by treating them as ‘assistants’, ‘helpers’, and ‘bystanders’ and placed their focus only on mothers and infants [39, 41, 46]. Conflicting and contradictory information given by family members and nurses were found [39, 46, 48]. Additionally, fathers found that the informational support they received on infant care was insufficient [39, 43, 46–48], which resulted in readmissions that were unwarranted [41]. Fathers reported that it is crucial to provide sufficient informational support to fathers during the hospital stay after child birth in order to avoid unnecessary readmission. Additionally, the information should be standardized across the various healthcare professionals to prevent confusion.

Change in a father’s behavior from self-oriented to family-oriented was seen as a process of change of becoming a father. Fathers sacrificed their personal, social, and leisure time for family time [44, 45] and put aside their feelings and sexual need to focus on the needs of their spouses and infants [41, 43, 48]. Fathering responsibility and parenting satisfaction were developed when they took care of their infants and their spouses during hospital stays [38, 39]. Feelings of wanting to protect their infants grew over time as fathers became closer with their infants [36, 37]. Feelings shaped fathers’ actual behaviors and it was important for fathers to attain parenting satisfaction for their behaviors to be actualized. Parenting satisfaction was found to have the largest direct effect on paternal involvement [53]. Therefore, healthcare professionals should aim to increase parenting satisfaction and fathering responsibility during hospital stays through encouraging direct contacts of a father and an infant, such as skin-to-skin contact, which fathers reported to be very good experiences [38].

### Implications for future research and practice

More qualitative research focusing exclusively on fathers is needed across geographical contexts throughout the perinatal period, especially in Asia and Africa where studies on fathering during infancy are lacking. Longitudinal quantitative research is needed to examine fathers’ involvement beyond one year. The effectiveness of improving father-infant relationships through skin-to-skin contact can be researched in different sociocultural contexts. Fathers should be prepared with infant care skills and knowledge before the birth of their infants so that they are prepared for postpartum challenges. The viability of imparting infant care skills and knowledge through online platforms can be explored as fathers are less likely to attend antenatal classes due to their work commitments.

### Limitation of the synthesis

The included studies in this meta-synthesis were mostly conducted in Western contexts, with only two studies conducted in Asia and one in Africa. Therefore, the findings may not be representative in understanding the experiences and needs of the fathers in other populations. Relevant studies might have been left out due to the exclusion of studies that were not published in English and due to unclear titles and abstracts and poor indexing.

## Conclusion

Fatherhood is shaped by sociocultural contexts and is influenced by multiple determinants. Across geographical regions, the father-infant relationship is heavily influenced by a father’s relationships with his spouse and family members. Reinforcements to involvement include father-infant bonding, fathers’ relationships with their own fathers, and spousal support. Work commitments were found to be the common hindrance to fathers’ involvement across regions. Additionally, healthcare professionals were found to exclude fathers during hospital stays where they were treated as ‘practical guys’ and ‘bystanders’ in Western and Singaporean contexts. The fathering experiences of fathers in the West differs from fathers in Asia due to differences in sociocultural contexts. Fathers in Singapore were socially not expected to be involved. The role of healthcare providers was emphasized in this meta-synthesis to play a part in encouraging fathers to be involved in the care of their infants during the early postpartum period.

## Supporting information

S1 Table PRISMA 2009 checklist. (DOC)

S2 Table Example of a search strategy. (DOCX)

S3 Table Results of critical appraisal of the included studies. (DOCX)

## References

[1] Lamb ME. Introduction: The Emergent American Father In: Lamb ME, redakteur. The Father Role: A Cross-cultural Perspective. Hillsdale, NJ: Lawrence Erlbaum Associates, Inc; 1987 bl 1–26.

[2] Barclay L, Lupton D. The experiences of new fatherhood: a socio-cultural analysis. J Adv Nurs. 1999;29:1013–20. 10215995 10.1046/j.1365-2648.1999.00978.x

[3] O’Brien M, Shemilt I. Working Fathers: Earning and Caring. Equal Opportunities Commission. 2003 http://www.fatherhoodinstitute.org/uploads/publications/280.pdf.

[4] LaRossa R. Fatherhood and Social Change. Fam Relat. 1988;37:451–7.

[5] Pleck JH. American Fathering in a Historical Perspective In: Kimmel MS, redakteur. Changing Men: New Directions in Research on Men and Masculinity. Newsbury Park, CA: SAGE Publications; 1987 bl 83–97.

[6] Giddens A. The Third Way. Cambridge, England: Polity Press; 1998.

[7] Beck U. Risk society: towards a new modernity. London, U.K.: Sage Publications; 1992.

[8] Beck U. The Reinvention of Politics: Towards a Theory of Reflexive Modernisation. In: Beck U, Giddens A, Lash S, redakteurs. Reflexive Modernisation Cambridge: Polity Press; 1994 bl 1–55.

[9] Williams S. What is Fatherhood? Searching for the Reflexive Father. Sociology. 2008;42:487–502.

[10] Yeung WJ. Asian Fatherhood. J Fam Issues. 2013;34:141–58. 10.1177/0192513X12461133

[11] Shan CH, Hawkins R. Childcare and parenting practices in Singapore: A comparison of fathers’ and mothers’ involvement. J Trop Psychol. 2014;4:1–12.

[12] Isaac R, Annie IK, Prashanth HR. Parenting in India. In: Selin H, redakteur. Parenting Across Cultures: Childrearing, Motherhood and Fatherhood in Non-Western Cultures Netherlands: Springer; 2014 bl 39–45.

[13] Locke C, Hoa NTN, Tam NTT. Visiting Marriages and Remote Parenting: Changing Strategies of Rural–Urban Migrants to Hanoi, Vietnam. J Dev Stud. 2012;48:10–25.

[14] Bracey G, Montie JE, Xiang ZP, Schweinhart LJ. The IEA preliminary study: Findings and policy implications. Ypsilanti: High/Scope Educational Research Foundation; 2007.

[15] Warin J, Solomon Y, Lewis C, Langford W. Fathers, work and family life. London, U.K.: Family Policy Studies Centre; 1999 http://eprints.lancs.ac.uk/20888/. Toegang verkry 23 Mei 2018.

[16] Gaertner BM, Spinrad TL, Eisenberg N, Greving KA. Parental Childrearing Attitudes as Correlates of Father Involvement During Infancy. J Marriage Fam. 2007;69:962–76. 10.1111/j.1741-3737.2007.00424 18174913 PMC2174267

[17] Yeung WJ, Sandberg JF, Davis-Kean PE, Hofferth SL. Children’s Time With Fathers in Intact Families. J Marriage Fam. 2001;63:136–54. 10.1111/j.1741-3737.2001.00136.x

[18] Volling BL, Belsky J. Multiple Determinants of Father Involvement during Infancy in Dual-Earner and Single-Earner Families. J Marriage Fam. 1991;53:461–74.

[19] Floyd K, Morman MT. Affection received from fathers as a predictor of men’s affection with their own sons: Tests of the modeling and compensation hypotheses. Commun Monogr. 2000;67:347–61. 10.1080/03637750009376516

[20] Wong MS, Mangelsdorf SC, Brown GL, Neff C, Schoppe-Sullivan SJ. Parental beliefs, infant temperament, and marital quality: Associations with infant–mother and infant–father attachment. J Fam Psychol. 2009;23:828–38. 10.1037/a0016491 20001141 PMC4422497

[21] Falceto OG, Fernandes CL, Baratojo C, Giugliani ERJ. Factors associated with father involvement in infant care. Rev Saude Publica. 2008;42:1034–40. 19009160 10.1590/s0034-89102008000600009

[22] Nangle S, Kelley M, Fals-Stewart W, Levant R. Work and Family Variables as Related to Paternal Engagement, Responsibility, and Accessibility in Dual-Earner Couples with Young Children. Fathering. 2003;1:71–90.

[23] Johnson K. Maternal-infant bonding: a review of literature. Int J Childbirth Educ. 2013;28:17+.

[24] Baker B, McGrath JM. Maternal-infant synchrony: An integrated review of the literature. Neonatal, Paediatr Child Heal Nurs. 2011;14:2–13.

[25] Kim M, Kang S-K, Yee B, Shim S-Y, Chung M. Paternal involvement and early infant neurodevelopment: the mediation role of maternal parenting stress. BMC Pediatr. 2016;16:212 10.1186/s12887-016-0747-y 27955632 PMC5153858

[26] Opondo C, Redshaw M, Savage-McGlynn E, Quigley MA. Father involvement in early child-rearing and behavioural outcomes in their pre-adolescent children: evidence from the ALSPAC UK birth cohort. BMJ Open. 2016;6:e012034 10.1136/bmjopen-2016-012034 27879246 PMC5128840

[27] Hall E. From Fun and Excitement to Joy and Trouble. Scand J Caring Sci. 1995;9:171–9. 7569522 10.1111/j.1471-6712.1995.tb00408.x

[28] Goodman JH. Becoming an involved father of an infant. J Obstet Gynecol Neonatal Nurs. 2005;34:190–200. 10.1177/0884217505274581 15781596

[29] Jensen LA, Allen MN. Meta-Synthesis of Qualitative Findings. Qual Health Res. 1996;6:553–60. 10.1177/104973239600600407

[30] Moher D, Liberati A, Tetzlaff J, Altman DG. Preferred reporting items for systematic reviews and meta-analyses: The PRISMA statement. Int J Surg. 2010;8:336–41. 10.1016/j.ijsu.2010.02.007 20171303

[31] Critical Appraisal Skills Programme. CASP (Qualitative) Checklist. 2018. https://casp-uk.net/wp-content/uploads/2018/03/CASP-Qualitative-Checklist-Download.pdf. Toegang verkry 21 Mei 2018.

[32] Sandelowski M, Barroso J. Handbook for synthesizing qualitative research. New York, N.Y: Springer Publishing Company, Inc; 2007.

[33] Braun V, Clarke V. Using thematic analysis in psychology. Qual Res Psychol. 2006;3:77–101.

[34] Onwuegbuzie AJ. Effect Sizes in Qualitative Research: A Prolegomenon. Qual Quant. 2003;37:393–409.

[35] Sandelowski M, Barroso J, Voils CI. Using qualitative metasummary to synthesize qualitative and quantitative descriptive findings. Res Nurs Health. 2007;30:99–111. 10.1002/nur.20176 17243111 PMC2329806

[36] Anderson AM. The Father-Infant Relationship: Becoming Connected. J Spec Pediatr Nurs. 1996;1:83–92. 10.1111/j.1744-6155.1996.tb00005.x 8933480

[37] Anderson AM. Factors Influencing the Father-Infant Relationship. J Fam Nurs. 1996;2:306–24. 10.1177/107484079600200306

[38] Ayala A, Christensson K, Velandia M, Erlandsson K. Fathers’ care of the newborn infant after caesarean section in Chile: A qualitative study. Sex Reprod Healthc. 2016;8:75–81. 10.1016/j.srhc.2016.02.007 27179381

[39] de Montigny F, Lacharité C. Fathers’ perceptions of the immediate postpartal period. J Obstet Gynecol Neonatal Nurs. 2004;33:328–39. 15180196 10.1177/0884217504266012

[40] Fägerskiöld A. A change in life as experienced by first-time fathers. Scand J Caring Sci. 2008;22:64–71. 10.1111/j.1471-6712.2007.00585.x 18269424

[41] Feenstra MM, Nilsson I, Danbjørg DB. “Dad–a practical guy in the shadow”: Fathers’ experiences of their paternal role as a father during early discharge after birth and readmission of their newborns. Sex Reprod Healthc. 2018;15 11 2017:62–8. 10.1016/j.srhc.2017.11.006 29389503

[42] Gamble D, Morse JM. Fathers Of Breastfed Infants: Postponing and Types of Involvement. J Obstet Gynecol Neonatal Nurs. 1993;22:358–65. 10.1111/j.1552-6909.1993.tb01816.x 8410435

[43] Olsson A, Robertson E, Björklund A, Nissen E. Fatherhood in focus, sexual activity can wait: New fathers’ experience about sexual life after childbirth. Scand J Caring Sci. 2010;24:716–25. 10.1111/j.1471-6712.2009.00768.x 20409069

[44] Premberg Å, Hellström AL, Berg M. Experiences of the first year as father. Scand J Caring Sci. 2008;22:56–63. 10.1111/j.1471-6712.2007.00584.x 18269423

[45] John WS, Cameron C, McVeigh C. Meeting the Challenge of New Fatherhood During the Early Weeks. J Obstet Gynecol Neonatal Nurs. 2005;34:180–9. 10.1177/0884217505274699 15781595

[46] Shorey S, Dennis C-L, Bridge S, Chong YS, Holroyd E, He H-G. First-time fathers’ postnatal experiences and support needs: A descriptive qualitative study. J Adv Nurs. 2017;73:2987–96. 10.1111/jan.13349 28557020

[47] Shorey S, Ang L, Goh ECL. Lived experiences of Asian fathers during the early postpartum period: Insights from qualitative inquiry. Midwifery. 2018;60:30–5. 10.1016/j.midw.2018.02.009 29477963

[48] Mbekenga CK, Lugina HI, Christensson K, Olsson P. Postpartum experiences of first-time fathers in a Tanzanian suburb: A qualitative interview study. Midwifery. 2011;27:174–80. 10.1016/j.midw.2009.03.002 20385433

[49] Shorey S, He H-G, Morelius E. Skin-to-skin contact by fathers and the impact on infant and paternal outcomes: an integrative review. Midwifery. 2016;40:207–17. 10.1016/j.midw.2016.07.007 27476026

[50] Cowan P, Cowan C. How working with couples fosters children’s development: From prevention science to public policy. In: Schulz MS, Pruett MK, Kerig PK, Parke RD, redakteurs. Strengthening couple relationships for optimal child development Washington, D.C: APA Publications; 2009 bl 211–28.

[51] Kitzmann KM. Effects of marital conflict on subsequent triadic family interactions and parenting. Dev Psychol. 2000;36:3–13. 10.1037//0012-1649.36.1.3 10645740

[52] Diemer GA. Expectant Fathers: Influence of Perinatal Education on Stress, Coping, and Spousal Relations. Res Nurs Heal. 1997;20:281–93. 10.1002/(sici)1098-240x(199708)20:4<281::aid-nur2>3.0.co;2-c 9256875

[53] Baker PJ. Self-efficacy, co-parenting relationship, and parent satisfaction: Variables that predict paternal involvement by non-custodial fathers. University of Pittsburgh, Pennsylvania, U.S; 2007.

