# Supplementary material for: Experiences, needs, and perceptions of paternal involvement during the first year after their infants’ birth: A meta-synthesis
Source: PLoS One. 2019 Jan 7;14(1):e0210388. doi: 10.1371/journal.pone.0210388 (PMC6322761; doi:10.1371/journal.pone.0210388)
Supplement: S2 Table — (DOCX) [file pone.0210388.s002.docx]

S2 Table: A draft of the search strategy conducted in one of the electronic database: PubMed

| Recent queries in pubmed | | | | | |
| --- | --- | --- | --- | --- | --- |
| Search | Add to builder | Query | Items found | Time |  |
| #6 | Add | Search (((((((((father*[Title/Abstract]) OR fathers[MeSH Terms]) OR paternal*[Title/Abstract]) OR husband*[Title/Abstract])) AND ((((((((postpartum*[Title/Abstract]) OR postpartal*[Title/Abstract]) OR postnatal*[Title/Abstract]) OR Postpartum Period[MeSH Terms]) OR Puerperium[MeSH Terms]) OR Parturition[MeSH Terms]) OR child birth*[Title/Abstract]) OR childbirth*[Title/Abstract])) AND ((experience*[Title/Abstract]) OR need*[Title/Abstract])) AND ((((((((((((((((((((((((infant care[Title/Abstract]) OR infant duties[Title/Abstract]) OR postpartum care[Title/Abstract]) OR Postnatal Care[MeSH Terms]) OR child care*[Title/Abstract]) OR childcare*[Title/Abstract]) OR child rearing[Title/Abstract]) OR involve*[Title/Abstract]) OR engage*[Title/Abstract]) OR interact*[Title/Abstract]) OR play*[Title/Abstract]) OR play behavior*[Title/Abstract]) OR bath*[Title/Abstract]) OR shower*[Title/Abstract]) OR breastfeed*[Title/Abstract]) OR breast feed*[Title/Abstract]) OR bond*[Title/Abstract]) OR coo*[Title/Abstract]) OR infant feed*[Title/Abstract]) OR talk*[Title/Abstract]) OR father-infant*[Title/Abstract]) OR skin-to-skin[Title/Abstract]) OR kangaroo care*[Title/Abstract]) OR infant diaper*[Title/Abstract]))) NOT mother*[Title/Abstract] | 198 | 22:13:02 |  |
| #5 | Add | Search (((((((father*[Title/Abstract]) OR fathers[MeSH Terms]) OR paternal*[Title/Abstract]) OR husband*[Title/Abstract])) AND ((((((((postpartum*[Title/Abstract]) OR postpartal*[Title/Abstract]) OR postnatal*[Title/Abstract]) OR Postpartum Period[MeSH Terms]) OR Puerperium[MeSH Terms]) OR Parturition[MeSH Terms]) OR child birth*[Title/Abstract]) OR childbirth*[Title/Abstract])) AND ((experience*[Title/Abstract]) OR need*[Title/Abstract])) AND ((((((((((((((((((((((((infant care[Title/Abstract]) OR infant duties[Title/Abstract]) OR postpartum care[Title/Abstract]) OR Postnatal Care[MeSH Terms]) OR child care*[Title/Abstract]) OR childcare*[Title/Abstract]) OR child rearing[Title/Abstract]) OR involve*[Title/Abstract]) OR engage*[Title/Abstract]) OR interact*[Title/Abstract]) OR play*[Title/Abstract]) OR play behavior*[Title/Abstract]) OR bath*[Title/Abstract]) OR shower*[Title/Abstract]) OR breastfeed*[Title/Abstract]) OR breast feed*[Title/Abstract]) OR bond*[Title/Abstract]) OR coo*[Title/Abstract]) OR infant feed*[Title/Abstract]) OR talk*[Title/Abstract]) OR father-infant*[Title/Abstract]) OR skin-to-skin[Title/Abstract]) OR kangaroo care*[Title/Abstract]) OR infant diaper*[Title/Abstract]) | 554 | 22:12:39 |  |
| #4 | Add | Search (((((((((((((((((((((((infant care[Title/Abstract]) OR infant duties[Title/Abstract]) OR postpartum care[Title/Abstract]) OR Postnatal Care[MeSH Terms]) OR child care*[Title/Abstract]) OR childcare*[Title/Abstract]) OR child rearing[Title/Abstract]) OR involve*[Title/Abstract]) OR engage*[Title/Abstract]) OR interact*[Title/Abstract]) OR play*[Title/Abstract]) OR play behavior*[Title/Abstract]) OR bath*[Title/Abstract]) OR shower*[Title/Abstract]) OR breastfeed*[Title/Abstract]) OR breast feed*[Title/Abstract]) OR bond*[Title/Abstract]) OR coo*[Title/Abstract]) OR infant feed*[Title/Abstract]) OR talk*[Title/Abstract]) OR father-infant*[Title/Abstract]) OR skin-to-skin[Title/Abstract]) OR kangaroo care*[Title/Abstract]) OR infant diaper*[Title/Abstract] | 4044517 | 22:12:19 |  |
| #3 | Add | Search (experience*[Title/Abstract]) OR need*[Title/Abstract] | 2291428 | 22:12:13 |  |
| #2 | Add | Search (((((((postpartum*[Title/Abstract]) OR postpartal*[Title/Abstract]) OR postnatal*[Title/Abstract]) OR Postpartum Period[MeSH Terms]) OR Puerperium[MeSH Terms]) OR Parturition[MeSH Terms]) OR child birth*[Title/Abstract]) OR childbirth*[Title/Abstract] | 268198 | 22:12:08 |  |
| #1 | Add | Search (((father*[Title/Abstract]) OR fathers[MeSH Terms]) OR paternal*[Title/Abstract]) OR husband*[Title/Abstract] | 72855 | 22:11:59 |  |
