# Supplementary material for: Experiences, needs, and perceptions of paternal involvement during the first year after their infants’ birth: A meta-synthesis
Source: PLoS One. 2019 Jan 7;14(1):e0210388. doi: 10.1371/journal.pone.0210388 (PMC6322761; doi:10.1371/journal.pone.0210388)
Supplement: S3 Table — (DOCX) [file pone.0210388.s003.docx]

S3 Table: Results of the critical appraisal of the included studies by the two independent authors using the Critical Appraisal Skills Programme tool with 10 criteria (Y = yes; N = no; CT = can’t tell)

| **Study** | **Author 1 (SS)** | | | | | | | | | |  | | **Author 2 (LA)** | | | | | | | | | |
| --- | --- | --- | --- | --- | --- | --- | --- | --- | --- | --- | --- | --- | --- | --- | --- | --- | --- | --- | --- | --- | --- | --- |
|  | **Q (Question)** | | | | | | | | | | | | | | | | | | | | | |
|  | **1** | **2** | **3** | **4** | **5** | **6** | **7** | **8** | **9** | **10** | | **1** | | **2** | **3** | **4** | **5** | **6** | **7** | **8** | **9** | **10** |
| 1. Anderson (1999a) | Y | Y | Y | Y | Y | Y | Y | Y | Y | Y | | Y | | Y | Y | Y | Y | Y | Y | Y | Y | Y |
| 1. Anderson (1999b) | Y | Y | Y | Y | Y | Y | Y | Y | Y | Y | | Y | | Y | Y | Y | Y | Y | Y | Y | Y | Y |
| 1. Ayala (2016) | Y | Y | Y | Y | Y | CT | Y | Y | Y | Y | | Y | | Y | Y | Y | Y | CT | Y | Y | Y | Y |
| 1. de Montigny and Lacharité (2004) | Y | Y | Y | Y | Y | CT | Y | Y | Y | Y | | Y | | Y | Y | Y | Y | Y | Y | Y | Y | Y |
| 1. Fagerskiold (2008) | Y | Y | Y | Y | Y | CT | Y | Y | Y | Y | | Y | | Y | Y | Y | Y | CT | Y | Y | Y | Y |
| 1. Feenstra et al. (2018) | Y | Y | Y | Y | Y | CT | Y | Y | Y | Y | | Y | | Y | Y | Y | Y | CT | Y | Y | Y | Y |
| 1. Gamble and Morse (1993) | Y | Y | Y | Y | Y | CT | Y | Y | Y | Y | | Y | | Y | Y | Y | Y | CT | Y | CT | Y | Y |
| 1. John et al. (2005) | Y | Y | Y | Y | Y | Y | Y | Y | Y | Y | | Y | | Y | Y | Y | Y | Y | Y | Y | Y | Y |
| 1. Mbekenga et al. (2011) | Y | Y | Y | Y | Y | Y | Y | Y | Y | Y | | Y | | Y | Y | Y | Y | Y | Y | Y | Y | Y |
| 1. Olsson et al. (2010) | Y | Y | Y | Y | Y | CT | Y | Y | Y | Y | | Y | | Y | Y | Y | Y | Y | Y | Y | Y | Y |
| 1. Premberg et al. (2008) | Y | Y | Y | Y | Y | CT | Y | Y | Y | Y | | Y | | Y | Y | Y | Y | CT | Y | Y | Y | Y |
| 1. Shorey et al. (2017) | Y | Y | Y | Y | Y | CT | Y | Y | Y | Y | | Y | | Y | Y | Y | Y | CT | Y | Y | Y | Y |
| 1. Shorey et al. (2018) | Y | Y | Y | Y | Y | Y | Y | Y | Y | Y | | Y | | Y | Y | Y | Y | CT | Y | Y | Y | Y |

Q1 Was there a clear statement of the aims of the research? Q2 Is a qualitative methodology appropriate? Q3 Was the research design appropriate to address the aims of the research? Q4 Was the recruitment strategy appropriate to the aims of the research? Q5 Was the data collected in a way that addressed the research issue? Q6 Has the relationship between the researcher and participants been adequately considered? Q7 Have ethical issues been taken into consideration? Q8 Was the data analysis sufficiently rigorous? Q9 Is there a clear statement of findings? Q10 How valuable is the research?
